# The Effect of Diesel Exhaust Particles on Adipose Tissue Mitochondrial Function and Inflammatory Status

**DOI:** 10.3390/ijms25084322

**Published:** 2024-04-13

**Authors:** Cali E. Warren, Kennedy M. Campbell, Madison N. Kirkham, Erin R. Saito, Nicole P. Remund, Kevin B. Cayabyab, Iris J. Kim, Micah S. Heimuli, Paul R. Reynolds, Juan A. Arroyo, Benjamin T. Bikman

**Affiliations:** Department of Cell Biology and Physiology, Brigham Young University, Provo, UT 84602, USA

**Keywords:** environmental pollution, systemic inflammation, mitochondrial dysfunction, adipose tissue dysfunction

## Abstract

Air pollution poses a significant global health risk, with fine particulate matter (PM_2.5_) such as diesel exhaust particles (DEPs) being of particular concern due to their potential to drive systemic toxicities through bloodstream infiltration. The association between PM_2.5_ exposure and an increased prevalence of metabolic disorders, including obesity, metabolic syndrome, and type 2 diabetes mellitus (T2DM), is evident against a backdrop of rising global obesity and poor metabolic health. This paper examines the role of adipose tissue in mediating the effects of PM_2.5_ on metabolic health. Adipose tissue, beyond its energy storage function, is responsive to inhaled noxious stimuli, thus disrupting metabolic homeostasis and responding to particulate exposure with pro-inflammatory cytokine release, contributing to systemic inflammation. The purpose of this study was to characterize the metabolic response of adipose tissue in mice exposed to either DEPs or room air (RA), exploring both the adipokine profile and mitochondrial bioenergetics. In addition to a slight change in fat mass and a robust shift in adipocyte hypertrophy in the DEP-exposed animals, we found significant changes in adipose mitochondrial bioenergetics. Furthermore, the DEP-exposed animals had a significantly higher expression of adipose inflammatory markers compared with the adipose from RA-exposed mice. Despite the nearly exclusive focus on dietary factors in an effort to better understand metabolic health, these results highlight the novel role of environmental factors that may contribute to the growing global burden of poor metabolic health.

## 1. Introduction

Air pollution is an increasingly grave concern globally, giving rise to myriad health complications worldwide. In 2019, the World Health Organization (WHO) estimated ambient air pollution causes 4.2 million premature deaths worldwide annually with 99% of the global population living in areas where the air quality standards recommended by WHO were unmet [1]. A particular concern within the context of air pollution is the elevated threat posed by fine particulate matter with an aerodynamic diameter of <2.5 μm (PM_2.5_) [2,3]. One of the most prevalent environmental pollutants is diesel exhaust particles (DEPs), constituting a common form of PM_2.5_ and presenting a daily exposure risk as a result of widespread vehicle emissions [4]. PM_2.5_ exhibits a notably large surface area-to-volume ratio that facilitates the absorption and accumulation of other toxic elements [5]. Consequently, the potential for PM_2.5_ to infiltrate the bloodstream through the lungs drives systemic toxicities.

While the detrimental effects of air pollution on certain health conditions such as chronic obstructive pulmonary disease and heart failure are widely recognized, a less evident yet equally relevant connection is the association between PM_2.5_ exposure and metabolic disorders. The growing incidences of metabolic disorders, including obesity, metabolic syndrome, and type 2 diabetes mellitus (T2DM), are particularly concerning as they coincide with a growing population residing in areas with unhealthy air quality [6]. In the United States, the prevalence of obesity affects more than two in five adults, which is approximately 42.5% of the population [7]. The scale of metabolic health concern is further emphasized by the estimation that 88% of U.S. adults are metabolically unhealthy, displaying at least one feature of metabolic syndrome [8]. Alarmingly, these statistics tend to be similar in countries throughout the world [9].

These troubling trends in metabolic disorders align with a growing body of research consistently indicating a positive correlation between the increase in metabolic disorders and PM_2.5_ exposure. Previous studies consistently associate PM_2.5_ exposure with obesity [10,11], reduced insulin sensitivity [12,13,14], and an elevated risk of T2DM [15,16]. However, these studies are limited to their reliance on correlation, leaving the specific causative factors driving these associations yet to be thoroughly investigated.

Considering its central role in metabolic disorders, it is not surprising that adipose tissue emerges as a pivotal mediator in understanding the intricate relationship between particulate matter and metabolic disorders. Beyond its conventional role in energy storage, adipose tissue may serve as a potential reservoir for the storage of pollutants, leading to adipose tissue dysfunction and the disruption of metabolic homeostasis [17,18]. Adipose tissue is known to become highly stimulated upon insult with noxious particulates, responding with a robust release of adipokines, including pro-inflammatory cytokines. While investigations into the impact of DEPs on metabolic health have previously revealed a connection with systemic inflammation [19], the purpose of our study was to characterize the metabolic response of adipose tissue to DEPs, including not only a characterization of the adipokine profile but also assessments of mitochondrial bioenergetics, which remains highly novel within the context of understanding the pollution–adipose concern.

## 2. Results

### 2.1. Body and Inguinal Adipose Tissue Weights

**Figure 1:**
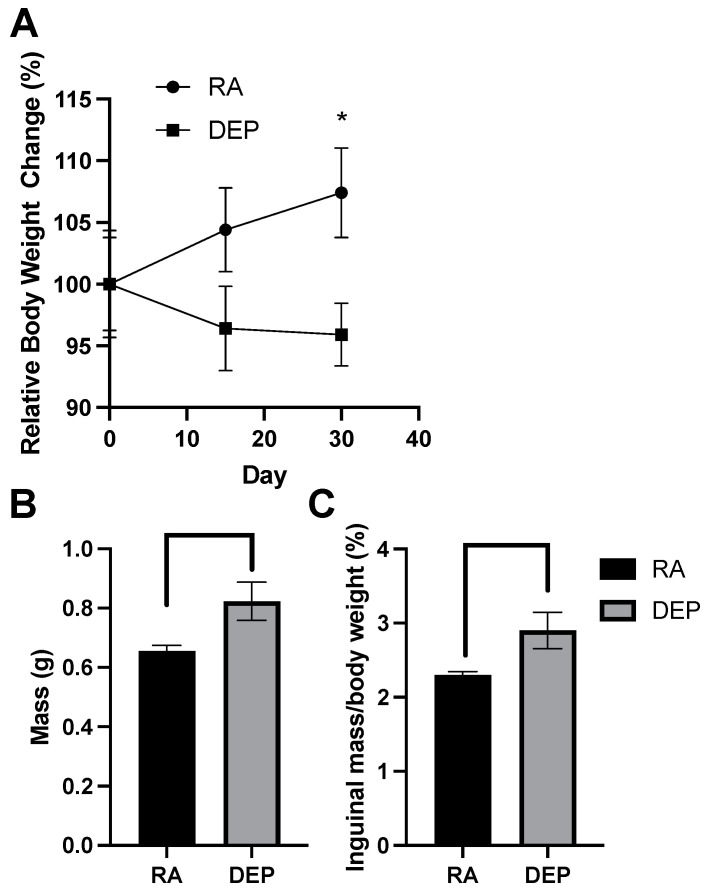
Diesel exhaust particle (DEP) vs. room air (RA) exposure alters body and inguinal adipose tissue weights. (**A**) Relative body weight change (%) of DEP and RA mice over a 30-day exposure period. (**B**) Total inguinal fat mass (g) extracted from DEP- and RA-exposed mice. (**C**) Inguinal mass/total body weight (%) ratios were calculated (* *p* < 0.05).

After recording the body weights on exposure days 0, 15, and 30, we assessed relative weight change over the 30-day period. A significant difference in relative weight change between diesel exhaust particle (DEP)-exposed mice and room air (RA) control mice was detected on day 30 of the exposure period (Figure 1A). Compared to the initial day 0 body weights, DEP-exposed mice had an overall decrease in body weight on day 30 while RA control mice had an increase in body weight. The difference between the relative weight changes in these two groups was significant (*p* < 0.05). Inguinal adipose tissue mass significantly increased in mice exposed to the DEP compared to the control (Figure 1B). The ratio of inguinal mass to body weight was calculated (Figure 1C), and DEP-exposed mice had significantly higher inguinal fat–body weight ratios compared to those of RA mice.

### 2.2. Mitochondrial Respiration

**Figure 2:**
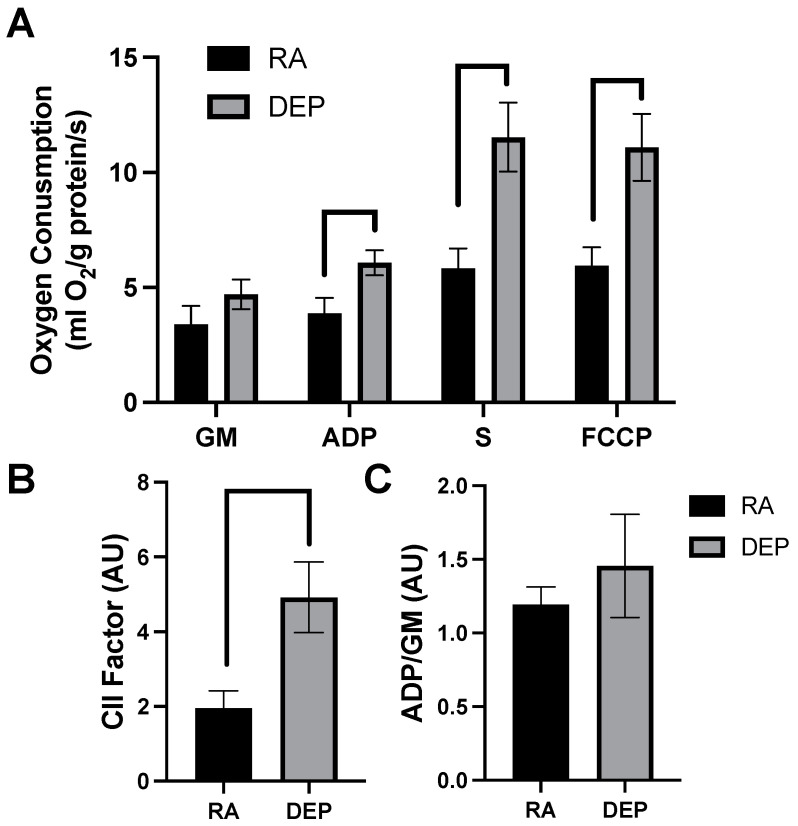
Diesel exhaust particle (DEP) vs. room air (RA) exposure alters mitochondrial oxygen consumption in inguinal adipose tissue. (**A**) Rates of mitochondrial oxygen consumption were measured via a substrate–uncoupler–inhibitor–titration protocol from inguinal adipose tissue following a 30-day exposure to RA or DEPs. The protocol included the addition of glutamate and malate (GM), ADP, succinate (S), and carbonyl cyanide 4- (trifluoromethoxy) phenylhydrazone (FCCP). (**B**) Respiratory control ratio (RCR) and (**C**) complex II-associated respiratory flux (CII factor) were calculated.

High-resolution respirometry indicated that inguinal adipose exposed to DEPs exhibited a significant increased rate of respiration compared to room air control adipose tissue. The significant elevations in mitochondrial respiration rate only occurred after the addition of ADP and continued with the addition of succinate (S) and carbonyl cyanide 4-trifluoromethoxy phenylhydrazone (FCCP; Figure 2A). Despite the difference in respiration rates observed in DEP-exposed mice, there was no significant difference in respiratory control ratios (RCRs), a general indicator of mitochondrial fitness, between treatments (Figure 2B). Complex II-associated respiratory flux (CII factor), a measure of complex II’s contribution to respiration rate, was significantly increased in DEP-exposed mice compared to room air ones (Figure 2C).

### 2.3. Inflammatory Marker Abundance following DEP Exposure

**Figure 3:**
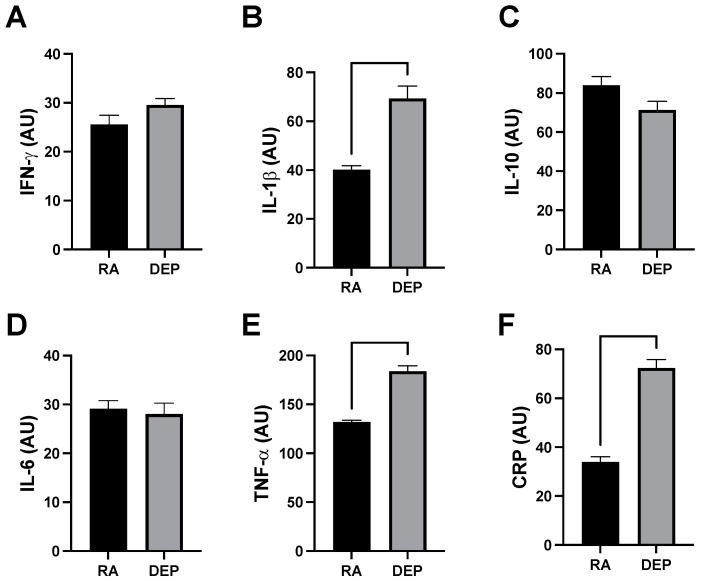
Diesel exhaust particle (DEP) vs. room air (RA) exposure increases pro-inflammatory markers in adipose tissue. (**A**–**F**) Notable inflammatory markers were screened in inguinal adipose tissue sample pools from RA and DEP mice. We discovered TNF-α, IL-1β, and CRP were significantly increased in DEPs compared to in RA.

Analysis of a mouse inflammation antibody array determined inguinal adipose in animals exposed to DEPs contained increased quantities of various cytokines. Specifically, we observed significant increases in the expression of tumor necrosis factor alpha (TNF-α), interleukin 1 beta (IL-1β), and C-reactive protein (CRP) (Figure 3A–C) from DEP-exposed adipose tissue sample pools compared to RA ones. We did not observe a significant difference in the cytokine interferon gamma (INFγ) (Figure 3D). Interleukin 6 (IL-6) and interleukin 10 (IL-10) were similarly unchanged between DEP and RA adipose (Figure 3E,F).

### 2.4. Adipokine Abundance following DEP Exposure

**Figure 4:**
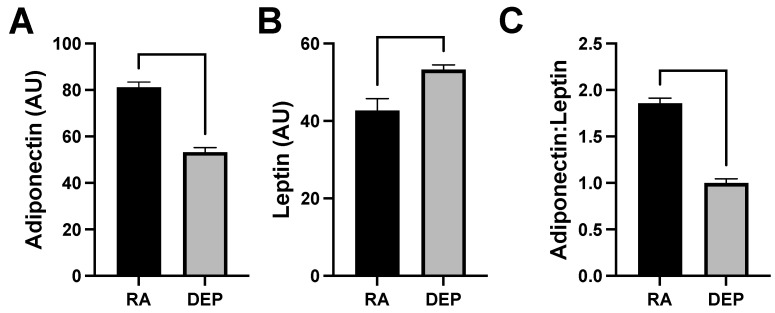
Diesel exhaust particle (DEP) vs. room air (RA) exposure decreases adiponectin and increase leptin levels in adipose tissue. (**A**,**B**) Levels of adiponectin and leptin were screened for and quantified in inguinal adipose tissue sample pools from DEP- and RA-exposed mice. (**C**) Adiponectin/leptin ratios were calculated.

Levels of adiponectin and leptin, which are cytokines specifically produced by adipose tissue (adipokines), were also evaluated in the mouse inflammation antibody array analysis. We observed a decrease in adiponectin levels (Figure 4A) and an increase in leptin (Figure 4B) in DEP adipose compared to in RA adipose. The ratio of adiponectin to leptin was calculated and utilized as a marker of adipose tissue inflammation and dysfunction. The adiponectin/leptin ratio decreased in adipose tissue samples exposed to DEPs compared to those to RA (Figure 4C).

### 2.5. Histological Analysis

**Figure 5:**
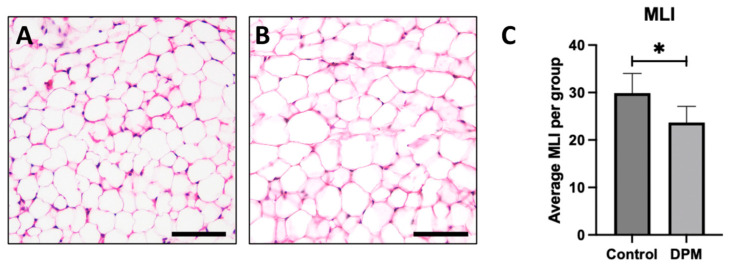
General adipocyte morphology and mean linear intercept. Representative adipose histology utilizing H&E staining of inguinal adipose biopsies revealed anticipated adipocyte morphology in control mice (**A**) and detectible larger adipocytes in DEP-treated mice (**B**). Morphological alterations were supported by assessing the mean linear intercept (MLI) of adipocyte boundaries wherein adipocytes from DEP-treated mice were larger than adipocytes from control (**C**). Images are representative of 6 randomized fields obtained. Scale bars represent 50 µm. (n = 6; * *p* < 0.05).

To investigate the impact of DEPs on adipocyte morphology, hematoxylin and eosin (H&E) staining was performed to observe general adipose morphology. Adipocytes from control mice exposed to room air revealed anticipated general morphology (Figure 5A) while larger adipocytes were observed in DEP-treated mice, indicating hypertrophic cell growth (Figure 5B). To further assess morphological changes, the mean linear intercept (MLI) measurements were quantified, and the average MLI in the DEP-exposed group was significantly decreased compared to in the RA control (Figure 5C).

## 3. Discussion

The coincident global rapid industrialization and occurrence of metabolic disorders suggests a relationship that is far more relevant than correlation. We have previously found that diesel exhaust particles (DEPs) are capable of aggravating lung macrophages, with a subsequent effect on the mitochondrial bioenergetics of these cells [20]. The purpose of this report was to redirect the focus to elucidate the specific effects of DEP exposure on adipose tissue mitochondrial function and adipokine production. Our novel findings provide an important molecular mechanism that facilitates our understanding of the consequences of inhaled particulates [21]. In particular, we have long known of the correlation between air pollution exposure and metabolic dysfunction, including type 2 diabetes (T2DM). Importantly, as we begin to better understand the etiology of T2DM and the essential role of insulin resistance, we more clearly see the role of both mitochondrial dysfunction and inflammation as contributing factors.

At the surface level, DEP exposure impacted body weight in a uniquely metabolically harmful direction, resulting in both reduced total body weight but increased subcutaneous adipose mass (Figure 2B,C). This change increased the subcutaneous fat as a percent of total body weight. Similar findings were noted in visceral adipose (results not shown). This could be a result of the DEP-induced activation of the receptor for advanced glycation end products, which has been shown to play a role in stimulating adipocyte hypertrophy [22].

Adipose hypertrophy is relevant beyond the simple expansion of adipose mass. Adipose contributes to metabolic disruption as each individual fat cell undergoes hypertrophy. With the expanded adipocyte volume, the cells become increasingly insulin-resistant and proinflammatory. Tragically, while each of these appears to be a mechanism to preserve adipocyte function by correcting excessive growth (i.e., insulin resistance) or hypoxia (i.e., inflammation), the unintended consequence throughout the body is a general increase in insulin resistance [23,24]. Given this effect, we sought to understand the effect of DEPs on adipocyte growth and whether some of the inflammation witnessed with DEP exposure might be a consequence of adipocyte hypertrophy. Histological sections of mouse adipose tissue revealed DEP adipocytes were larger in size than adipocytes from room air (RA) controls (Figure 5A,B). This distinguishable increase in adipocyte size was accompanied by a noteworthy reduction in mean linear intercept (MLI) measurements in DEP-exposed mice. Collectively, the increase in adipocyte size and decrease in MLI following exposure to DEPs demonstrated the impact of DEP-induced adipose hypertrophy. As adipocytes undergo hypertrophy, a cascade of metabolic disturbances ensues, giving rise to insulin resistance and proinflammatory responses.

As an additional marker of adipocyte growth, we analyzed the adiponectin and leptin levels. One quirk of adipocyte physiology is the shift in adipokine expression with adipocyte hypertrophy, and whereas leptin levels continue to climb as the adipocyte expands, adiponectin levels plateau or drop [25]. Thus, a reduction in the adiponectin/leptin ratio (Figure 4C) is further evidence of growth.

In addition to the mitochondrial response, adipose tissue manifested with a significant elevation in the production of pro-inflammatory cytokines (Figure 3), including c-reactive protein, interleukin-1β, and tumor necrosis factor-α. These results suggest that at least some of the robust inflammatory response observed systemically in response to DEP exposure is due to an adipose contribution.

Despite their low metabolic demand, adipocytes contain and rely on mitochondria for a number of processes, including energy, lipid metabolism, and more. We found that DEP exposure elicited a robust, two-fold increase in O_2_ consumption when all mitochondrial complexes were engaged (Figure 2A), particularly with inclusion of complex II (Figure 2B). Due to limitations with sample size, we were unable to measure oxidative stress in the tissues. However, based on our previous results [26], we suspect at least part of the increased respiration contributed to increased reactive oxygen species generation. Furthermore, there are additional notable limitations inherent to this work. Due to our use of whole adipose tissue, it is possible that our data inadvertently include results from stromal vascular cells (SVCs), a population of cells found within adipose tissue. Importantly, SVCs can be as much as 30% of the resident cell population within adipose tissue [27].

In an effort to address the ever-growing global burden of poor metabolic health, significant attention has classically focused on dietary variables. However, while undoubtedly vital, the results from this work suggest the importance of environmental factors that extend beyond food. Our findings provide a highly novel contribution to our understanding of the various stimuli, even non-caloric, that influence the metabolic status of adipose tissue. Additionally, with this insight comes the potential to address novel biochemical pathways and receptors that deal more with inhaled particles, rather than just nutrient- or caloric-based approaches. Ultimately, in addition to strategies focused on dietary adjustment, these results show that variables beyond diet should be part of a larger strategy to improve metabolic outcomes. 

## 4. Materials and Methods

### 4.1. Animals

Male and female C57BL/6 purchased from Jackson Laboratories (Bar Harbor, ME, USA) were housed and cared for in a standard pathogen-free facility at 22 ± 1 °C, 60–70% humidity on a 12 h light/dark cycle. Animals were given access to a standard chow diet and water ad libitum. Mice were randomly divided into control room air (RA)-exposed and treatment diesel exhaust particle (DEP)-exposed groups for six weeks. Room air was not measured for ambient DEPs. Body weights for the mice were recorded on days 0, 15, and 30 of the exposures. At the conclusion of the exposures, mice were sacrificed, and inguinal adipose tissue samples were precisely extracted and weighed. Freshly dissected inguinal adipose tissue samples were either fixed in 4% paraformaldehyde for histological analysis, snap-frozen in liquid nitrogen and stored at –80 °C for inflammatory marker/cytokine analysis or placed in saponin buffer for mitochondrial respirometry. Animal studies were approved by the Institutional Animal Care and Use Committee (IACUC) at Brigham Young University and conducted in accordance with the procedures and regulations outlined in the National Institutes of Health Guide for the Care and Use of Laboratory Animals.

### 4.2. Diesel Exhaust Particle Exposure

The DEP used in these mice experiments is cataloged at the National Institute of Standards and Technology (NIST) as Standard Reference Material (SRM) 2975. SRM-2975 was originally obtained from M.E. Wright of the Donaldson Company, Inc., Minneapolis, MN, USA. The DEP used to prepare SRM-2975 was collected from a filtering system designed specifically for diesel-powered forklifts [20]. Following collection, the DEP was homogenized and extracted for preparation of SRM-2975.

Mice were placed in soft restraints and connected to the exposure tower of a nose-only exposure system (InExpose System; Scireq, Montreal, QC, Canada). The DEP-exposed mice group received a nebulized dose of 15 ng of freshly vortexed DEPs in approximately 20 μL PBS. This translates to roughly 3 ug/mL in plasma, which is considered a physiological dosage [28]. DEP exposures lasted 30 min per day five days a week for six weeks for 30 exposures total. Control animals exposed to RA were similarly restrained and allowed to breathe room air.

### 4.3. Mitochondrial Respirometry

Mitochondrial oxygen consumption rates were determined at 37 °C from freshly isolated inguinal adipose tissue using the Oroboros O2K Oxygraph (Oroboros, Innsbruck, Austria) with MiR05 respiration buffer as described previously [19]. Before adipose samples were transferred to the respirometer chambers of the O2K Oxygraph, the tissue samples were permeabilized in 0.05 mg/mL of saponin (Sigma-Aldrich, St. Louis, MO, USA) in MiR05 for 30 min at 4 °C. After the addition of the permeabilized inguinal adipose tissue, the respirometer chambers were hyperoxygenated to ~350 nmol/mL. The chambers were then closed, and it took approximately 5 min for a stable baseline respiration rate to be established. Changes in oxygen consumption rates were then determined following a substrate–uncoupler–inhibitor–titration (SUIT) protocol. Electron flow through complex I was supported by the addition of glutamate + malate together (10 mM and 2 mM, respectively) to determine the leak oxygen consumption (GM). Following stabilization, adenosine diphosphate (ADP) (2.5 mM) was added to determine oxygen consumption associated with oxidative phosphorylation. Succinate (S) (10 mM) was added to support complex I + II electron flow into the Q-junction. Lastly, to obtain maximal uncoupled respiration, the chemical uncoupler carbonyl cyanide 4-trifluoromethoxy phenylhydrazone (FCCP) was titrated (steps of 0.05 Μm) to maximize electron transport capacity.

Following the conclusion of the respiration protocol, adipose tissue samples were collected from the chambers and stored at −20 °C for further analysis. Protein concentrations were measured via the BCA assay (Perkin Elmer, Waltham, MA, USA), and the respiration rates were normalized to the protein concentration. From the respiratory fluxes obtained during the respiration protocol, the respiratory control ratio (RCR) and complex II-associated respiratory flux (CII factor) were determined by calculating the ratio of ADP:GM and the difference between S and ADP, respectively.

### 4.4. Inflammatory Cytokine Analysis

Notable inflammatory markers were measured using a mouse inflammation antibody array (Abcam, Waltham, MA, USA). Previously snap-frozen inguinal adipose tissue was homogenized in protein lysis buffer provided in the antibody array kit supplemented with a protease and phosphatase inhibitor cocktail (Thermo Fisher Scientific, Pittsburg, PA, USA). The total protein in the lysates was measured using a BCA Protein Assay Kit (Thermo Fisher Scientific), and 25 μg of protein from five animals in each treatment group (RA or DEP) was pooled with a final concentration of 125 μg/mL. The array was performed as outlined in the manufacturer’s protocol. Briefly, the sample pools were added to membranes containing specific capture antibodies and incubated for two hours at room temperature before being incubated again with a second antibody array membrane. Next, biotin-conjugated antibodies were pipetted into each well and incubated. Lastly, streptavidin-HRP, a fluorescent label, was added to each membrane for a final incubation to detect inflammatory molecule expression. The cytokine array membranes were imaged using the Odyssey DLx Near-Infrared Fluorescence Imaging System (LI-COR) and quantified using Image J (U.S. National Institutes of Health, Bethesda, MD, USA) [29].

### 4.5. Histology

Following dissection, inguinal adipose tissue was fixed in 4% paraformaldehyde, processed with a series of ethanol washes, embedded in paraffin, and stored at 4 °C. The embedded adipose tissue blocks were then sectioned at 5µm thickness [30]. Hematoxylin and eosin (H&E) staining was performed to observe morphological changes in adipocytes and quantify adipocyte diameter. The slides were deparaffined and stained with hematoxylin and eosin (Thermo Scientific, Pittsburg, PA, USA) using standard techniques. Approximate adipocyte diameter was estimated via mean linear intercept (MLI) measurement, which is a common metric used to quantify the mean distance between landmarks [26,31]. In this case, morphological alterations were evaluated between DEP-exposed and control mice by MLI using a point-counting method specifically assessing adipocyte cell membrane intercepts. At least 6 images were obtained from each treatment group with an Olympus BX51 microscope using Olympus CellSense Standard 3.1 and intercepts counted from each inguinal fat biopsy were averaged.

### 4.6. Statistical Methods

Data are presented as the mean ± standard error of the mean (SEM). Differences between the RA and DEP means were compared using Student’s *t*-tests. GraphPad Prism software version 7.0 (GraphPad; Santa Clara, CA, USA) was used for statistical analyses. Statistical significance was set at *p* < 0.05.

## Data Availability

Data can be made available by contacting the corresponding author.
